# Changes in Oxidative Stress, Inflammation, and Muscle Damage Markers Following Diet and Beetroot Juice Supplementation in Elite Fencers

**DOI:** 10.3390/antiox9070571

**Published:** 2020-07-01

**Authors:** Lucyna Kozłowska, Olga Mizera, Jolanta Gromadzińska, Beata Janasik, Karolina Mikołajewska, Anna Mróz, Wojciech Wąsowicz

**Affiliations:** 1 Department of Dietetics, Warsaw University of Life Sciences, Institute of Human Nutrition Sciences, Nowoursynowska 15c, 02-776 Warsaw, Poland; lucyna_kozlowska@sggw.pl; 2 Department of Biological and Environmental Monitoring, Nofer Institute of Occupational Medicine, Sw. Teresy 8, 91-348 Łódź, Poland; jolanta.gromadzinska@imp.lodz.pl (J.G.); beata.janasik@imp.lodz.pl (B.J.); karolina.mikolajewska@imp.lodz.pl (K.M.); wojciech.wasowicz@imp.lodz.pl (W.W.); 3 Department of Physiology and Sport Medicine, Jozef Pilsudski University of Physical Education in Warsaw, Marymoncka 34, 00-968 Warsaw, Poland; anna.mroz@awf.edu.pl

**Keywords:** malondialdehyde, advanced oxidation protein products, 8oxodG, ceruloplasmin, glutathione peroxidase, lactate dehydrogenase, interleukin 6, β-carotene, maximum rate of oxygen uptake, trace elements

## Abstract

The aim of this study was to assess the impact of diet and active substances in beetroot juice on the parameters of oxidative stress, inflammation, and muscle damage as well as on the maximum rate of oxygen uptake (VO_2max_) in elite fencers (10 women, 10 men). Athletes during four weeks realized dietary recommendations (ID) and, after that, diet with freeze-dried beetroot juice supplementation (ID&BEET). At baseline and after each stage, fasting antioxidants, biomarkers of oxidative stress, inflammation, and skeletal muscle damage were measured, and a VO_2max_ test was performed. Only after ID&BEET was a significant increase of VO_2max_ observed, and changes of this parameter were negatively related with changes of serum lactate dehydrogenase (∆LDH) activity, as well as with serum ∆β-carotene and malondialdehyde concentration (∆MDA). Additionally, positive relationships were observed between ∆β-carotene versus changes of the serum concentration of advanced oxidation protein products (∆AOPP), changes of serum glutathione peroxidase activity (∆GPx3) versus both changes of physical activity level and ∆LDH, as well as erythrocyte glutathione peroxidase activity (∆GPx1) versus ∆LDH. To summarize, we showed that long-term beetroot juice supplementation increases lipid peroxidation, and improvement of VO_2max_ after ID&BEET seems to be dependent on LDH activity, as well as on the serum concentration of MDA and β-carotene.

## 1. Introduction

Beetroot (*Beta vulgaris var. rubra*) is a rich source of important phytochemical compounds such as ascorbic acid, carotenoids, phenolic acids, flavonoids [1,2,3], and a group of bioactive pigments known as betalains [4,5]. In many in vitro and in vivo animal models, it has been shown that betalains have high antioxidant and anti-inflammatory capabilities [5,6,7,8,9]. Moreover, highly bioactive phenolics such as caffeic acid, epicatechin, and rutin are known as excellent antioxidants [1,10]. Beetroot juice is also a rich source of inorganic nitrates (NO_3_^-^), which are a substrate for the synthesis of nitric oxide [11]. Nitrite and nitric oxide have been shown to suppress radical formation and to quench potentially damaging reactive oxygen (ROS) and nitrogen (RNS) species, which suggests that nitrate may also exhibit antioxidant effects [12,13,14]. Dietary nitrates are of interest due primarily to their potential for relaxing human vasculature [15], as this effect may improve blood flow during exercise [16,17]. Many studies have shown the ergogenic effect of chronic and acute supplementation of nitrate, connected to improvements in walking [18,19], running [20,21], rowing [22,23], cycling [18,24], submaximal exercise [20,25], and of tolerance to more vigorous exercise [18,20].

However, it is worth noting that not all studies have demonstrated a significant improvement in performance and blood flow following dietary nitrate ingestion [26,27,28,29,30,31,32]. The authors of these studies have put forward some hypotheses regarding the lack of an ergogenic effect. These include, among others, variability of individual responses among participants, supposition that high-trained elite athletes may require a higher amount of dietary nitrate, or that athletes are more resistant to supplementation, as well as changes in microbiota reductase activity, which is associated with impaired conversion of nitrate to nitrite [27,28]. It also seems that an important aspect able to determine the effectiveness of dietary nitrates may be the length of the period in which nitrates are supplemented—a long period of supplementation in conjunction with intense physical activity may intensify oxidative stress and may increase the risk of overtraining and muscle fatigue, which may consequently weaken the ergogenic effect of dietary nitrates.

Physical activity, associated with increased oxygen consumption, generates ROS production and may lead to oxidative stress. Ischaemia reperfusion [33,34], haemoglobin and myoglobin oxidation [35,36], increased central temperature, catecholamine, and lactic acid production are main processes involved in ROS production during exercise [33,37]. The biological effects of ROS can be both positive and negative. The positive effects include immunity, action as cell messengers, modification oxidation-reduction status, modification of signaling pathways as well as an increase in strength contraction [38,39]. Meanwhile, the negative effects of ROS include damages of important macromolecules in cells and extracellular fluids as well as apoptosis of healthy cells, inflammation, and a change in the cellular function alteration of the size and shape of compounds [33]. ROS production during mitochondrial respiratory chain in the muscles during physical activity modulates main cellular pathways for adaptation to exercise [40]. It has been shown that oxidative stress may be one of the main actors of muscular fatigue and overtraining syndrome [41,42]. ROS can oxide polyunsaturated fatty acids, which are an important component of cell membranes, blood, and structural proteins, and which can lose amino acids or become fragmented [43,44,45]. Therefore, it seems possible that ROS and RNS can induce a decrease in maximal force, and that repetitive muscular fatigue associated with inadequate recovery is supposed to induce overtraining syndrome [46,47].

However, there are no long-term studies in highly trained athletes that comprehensively analyze the various factors that may determine the net effect obtained by beetroot juice supplementation. Therefore, the aim of this study was to assess the impact of diet and active substances in beetroot juice on the parameters of oxidative stress, inflammation, and muscle damage as well as on the maximum rate of oxygen uptake in elite fencers.

## 2. Materials and Methods 

### 2.1. Participants and Study Design

The study included 24 elite fencers (12 men and 12 women). However, four people did not complete the study due to various reasons (minor injuries in two players, health problems in one player, and personal problems in one player). Finally, 10 women (age 22.6 ± 5.3 years) and 10 men (27.2 ± 5.4 years) completed the study. Each participant had been training in fencing for a minimum of three years, and at least three times a week. The exclusion criteria were as follows: The use of alternative diets, smoking, the occurrence of chronic diseases, taking dietary supplements or other ergogenic agents, as well as antibiotics and steroids or non-steroidal anti-inflammatory drugs—and, in the case of women, also irregular menstruation. The highly trained fencers were unable to refrain from exercising for a few days before each stage of the study, because this conflicted with their training schedules. After receiving a detailed explanation of the study protocol, each participant provided a written informed consent. The study was approved by the Ethics Committee of the Nofer Institute of Occupational Medicine Nr 12/2013.

The research program consisted of an initial data analysis (stage B) and two consecutive stages. In the first stage, which lasted four weeks, the dietary recommendations were implemented (stage ID), after which, in the second stage, also lasting four weeks, the same dietary recommendations were implemented; additionally, the participants received freeze-dried beetroot juice in the amount of 26 g per day, which corresponded to one glass of juice (200 ml) (stage ID&BEET). The preparation of the freeze-dried beetroot juice is described in our previous article [48]. The nutritional value of beetroot juice is in the Supplementary Materials (Table S1).

**Figure 1:**
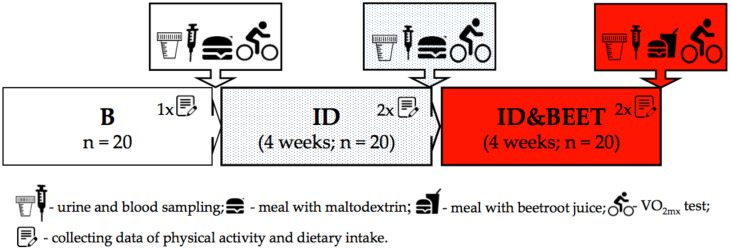
Study design. On the last day of each stage blood samples were collected and after a special meal, a maximum rate of oxygen uptake (VO_2max_) was conducted. B: The first stage of the study (baseline characteristic); ID: The second stage of the study (implementation of dietary recommendations without beetroot juice supplementation); ID&BEET: The third stage of the study (implementation of dietary recommendations with beetroot juice supplementation).

At stage B and at each stage thereafter, the fencers visited a laboratory after overnight fasting and were instructed to empty their bladder in order to collect a urine sample. Afterward, measures of body composition were performed and venous blood samples were taken. At stage B and two times at each stage thereafter (i.e., in the 2nd, 4th, 6th, and 8th weeks of the study), data pertaining to physical activity and dietary intake were collected. This information came from the following three days: A day that included fencing training, a day that included general training, and a day free from training. The research plan is shown in Figure 1.

Each laboratory visit was carried as follows:

1. After all-night fasting, the blood and urine samples were collected. Then, body composition was measured.
2. Afterward, a meal with a particular composition was consumed: Toasted bread (120 g), cold meats (sirloin, 60 g), banana (100 g), and a butter mix (24 g), as well as maltodextrin (26 g) or freeze-dried beetroot juice (26 g) dissolved in 150 ml of water. The nutritional value of this meal was as follows: Energy, 787 kcal; protein, 23 g; total fat, 27; total carbohydrate, 117 g. Before the physical test, the fencers consumed a meal with maltodextrin after the first and the second stages (B and ID), but after the third stage (ID&BEET), freeze-dried beetroot juice was added to the meal.

**Figure 2:**
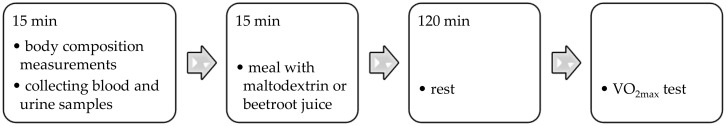
The protocol of laboratory measurements conducted in the group of female and male fencers after each stage of study. The fencers after the first and second stage (B, ID) of the study, before the maximum rate of oxygen uptake test (VO_2max_) consumed a meal with maltodextrin (26 g). Whereas after the third stage of the study (ID&BEET), fencers consumed a meal with freeze-dried beetroot juice (26 g)—both dissolved in water.

Two hours after the consumption of a meal, a maximum rate oxygen uptake test VO_2max_ was performed. The laboratory visit plan is shown in Figure 2.

Each participant was instructed to avoid using antibacterial mouthwashes during the supplementation of beetroot juice. The use of an antiseptic mouthwash with active antibacterial properties may affect nitrate conversion [49].

### 2.2. Anthropometric Parameters, Physical Activity Level, and Maximum Rate of Oxygen Uptake

After each stage (i.e., B, ID, and ID&BEET), anthropometric parameters were measured. Height was measured to the nearest 5 mm using a wall-mounted stadiometer. The weight of the fencers dressed light in underwear was measured to the nearest 10 g using a digital scale. Body composition (fat mass percent (FM%) and fat free mass percent (FFM%)) was measured using a single frequency bioimpedance analyzer (BC418MA; Tanita, Tokyo, Japan), operating at 50 kHz with eight-point contact electrodes. This analyzer consists of a platform with stainless-steel foot pads and two handgrips with stainless-steel contacts. The four contacts on the platform were arranged in a position of two contacts for each foot, and each handgrip contained one pair of contacts. The measurements were taken in a standing position, and each participant was measured in underwear. The measurements were performed according to the standard procedures [50].

Data on physical activity were collected from a three-day physical activity logbook and the energy cost of physical activities MET (Metabolic Equivalent of Task) manual [51]. For each participant at baseline and at two times at each stage (i.e., in the 2nd, 4th, 6th, and 8th weeks of the study), physical activity data were collected for 24 h on three following days: A day that included general development training, a day that included fencing training, and a training-free day. Due to the fact that there were no significant differences in the level of physical activity in the 2nd and 4th as well as in the 6th and 8th weeks, the data were averaged. Subsequently, in each stage, the weekly average (PA) was calculated. To calculate the weekly average, the following were taken: Four days that included fencing training, two days that included general development training, and a training-free day.

The maximum rate of oxygen uptake (VO_2max_) was measured using an incremental protocol [52] on the bicycle ergometer Ergoselect 200 Ergoline GmbH (Bitz, Baden-Württemberg, Germany) and the ergospirometer MES 2000 (Cracow, Poland). A detailed description of the methodology was described by us in an earlier article [45].

### 2.3. Nutritional Recommendations for Athletes

Before the therapy began, the athletes received dietary recommendations with individually calculated energy and nutrient values. The nutritional recommendations for the athletes included protein intake at a level of 1.5–2.0 g/kg body weight and fat at a level of 30% of total energy [53,54]. The total energy demand for each person was estimated during the baseline stage (B) on the basis of the filled-in physical activity logbook and the energy cost of physical activities manual (i.e., MET) [51]. The nutritional recommendations for the other nutrients referred to the dietary reference intake standards for the Polish population: Adequate Intake (AI) for vitamin E, potassium, and sodium; and the Recommended Dietary Allowance (RDA) for the other nutrients [55].

### 2.4. Dietary Records and Analysis of Dietary Intake

At baseline (B) and at two times at each stage thereafter (i.e., in the 2nd, 4th, 6th, and 8th weeks of the study), the athletes filled in the three-day (i.e., a day that included fencing training, a day that included general development training, and a training-free day) dietary record of consumed food and beverages. Each dietary record was verified by using an album of photographs of food products and dishes, which contained three suggested portion sizes. Based on collected data from the three-day dietary record of consumed food and beverages, the percent of fulfilling the norm and dietary recommendations for nutrients was determined. Due to the fact that there were no significant differences in the level of nutrient intake in the 2nd and 4th as well as in the 6th and 8th weeks, the data were averaged. Further analyses of the nutrients consumed were carried out between three averaged results: Those from stage B, those from the 2nd and 4th weeks of stage ID, and those from the 6th and 8th weeks of stage ID&BEET. Dietary energy intake is presented as kilocalories per kilogram body weight, and protein, fat, and carbohydrates are presented as percent of total energy. Three-day food records were analyzed for nutrient intake using the diet processor software—Dieta5 (Institute of Food and Nutrition, Warsaw, Poland). The nutrients, macronutrients, micronutrients, and vitamins were analyzed without the nutritional values of freeze-dried beetroot juice, which was supplemented in stage ID&BEET.

### 2.5. Blood Sampling and Measurements of Antioxidants, Biomarkers of Oxidative Stress, Inflammation, and Skeletal Muscle Damage 

Urine and blood samples were collected after 12 h of fasting. Venous blood was obtained with each subject in a seated position—2.5 mL venous blood samples were collected into heparinized anticoagulant S-MONOVETTE and 5.0 mL of blood was collected into SepClot Activator Vacuettes test tubes by means of cubital venipuncture. Blood was centrifuged, serum was collected into eppendorf tubes. Heparinized blood was centrifuged too, the plasma and buffy coat was rejected, erythrocytes were washed three times with 0.9% NaCl, and hemolyzed for freezing and thawing. The urine as well as the separated serum and washed hemolysates were immediately frozen at –80 °C until further analysis could be performed. Hemoglobin concentration in hemolysates was determined with the Drabkin reagent.

Serum vitamins A and E and beta-carotene levels were determined by use of the Waters 2695 Alliance LC System (Waters, Manchester, UK) integrated with a 190–800 nm UV–Vis detector [56]. NIST-968e (USA; fat-soluble vitamins and carotenoids in human serum) were used as a standard. 

For the serum selenium analysis, serum samples were diluted 150-fold with 1.0% HNO_3_. Concentrations of Se were determined using inductively coupled plasma mass spectrometry (ICP–MS; Elan DRC-e, Perkin Elmer, SCIEX, Norwalk, CT, USA) [57]. The accuracy of the method was verified using an internal quality control of the certified reference material BCR-637 (Institute for Reference Materials and Measurements, Geel, Belgium), where the reference value and the measured concentration were 81.0 µg/L (range 74–88 µg/L) and 82.5 ± 0.7 µg/L, respectively. With regard to the determination of Se in the biological samples, the laboratory in charge of it participated in the German External Quality Control (G-EQUAS) organized by the Institute of Occupational Social and Environmental Medicine of the University of Erlangen, Nuremberg.

For serum copper and zinc analyses, serum samples were diluted 30-fold with 1.0% HNO_3_. Concentrations of Cu and Zn were analyzed by ICP–MS (Elan DRC-e, Perkin Elmer, SCIEX, Norwalk, CT, USA). The accuracy of the method was verified using an internal quality control of the certified reference material BCR-637 (Institute for Reference Materials and Measurements, Geel, Belgium) for zinc, where the reference value and the measured concentration were 1110.0 µg/L (range 890–1330 µg/L) and 1010 ± 11 µg/L, respectively. For Cu, the method was validated using reference material (lyophilized human reference serum samples of Clincheck^®^, Recipe, GmbH, Germany).

The oxidase activity of ceruloplasmin (CP) in the serum was measured with p-phenyldiamine as a substrate according to the method described by Sunderman and Nomoto [58]. Absorbance of the substrate oxidation product was measured at 535 nm using the Evolution UV–Vis spectrophotometer (Thermo Scientific, Waltham, MA, USA).

In hemolyzed erythrocytes (GPx) and serum GPx, activities were assayed according to the method of Paglia and Valentine [59] with t-butyl hydroperoxide as a substrate. The GPx activities was measured spectrophotometrically by decreasing absorbance at 340 nm during NADPH oxidation to NADP+, using the Evolution UV–Vis spectrophotometer. The enzyme activities are expressed in units per gram of hemoglobin (GPx1) or milliliters of serum (GPx3) at 25 °C. The intra-assay coefficient of variability (CV) amounted to 5%.

The advanced oxidation protein products (AOPP) in serum were measured using the immunoassay technique (Cloud-Clone Corp CEB223Hu, USA). The measurements were quantified using the standard spectrophotometer (Multiscan GO; Thermo Scientific, Waltham, MA, USA).

Malondialdehyde (MDA) in serum was determined in one isocratic high-performance liquid chromatograph (HPLC) run with fluorescence detection using a Chromsystems kit. The sample preparation was based on an effective protein precipitation step, followed by derivatization. The resulting fluorophore was specific and detectable at wavelength excitation of 515 nm, and an emission of 553 nm. The measurements were performed by the Waters 2695 Alliance LC System (Waters, Manchester, UK).

The concentration of 8-oxo-7.8-dihydro-2′-deoxyguanosine (8-oxodG) in urine was quantified using the HPLC Waters 2695 Alliance LC System (Waters, Manchester, UK). The HPLC separations module was coupled with MS/MS detection (tandem mass spectrometer) Micromass Quattro Micro API (Waters, Milford, MA, USA), equipped with an electrospray ionization (ESI) interface. The collected urinary samples were analyzed by adding 0.06 nmol of internal standard [15N5]-8-hydroxy-2’-deoxyguanosine (Cambridge Isotope Laboratories, Inc., Andover, MA, USA) and 0.75 mL of water to 0.25 mL of urine. The samples were purified on a syringe filter (Millex-FG Filter, Fluoropore from Merck Millipore, St. Charles, MO, USA). Then, 10 μL of the extract was injected into the analytical column Kinetex 2,6u XB-C18 100A 50 × 2.1 mm (Phenomenex). The elution was carried out with the following composition: Water with 0.4% of solvent A, acetic acid (Sigma-Aldrich) in water and solvent B, acetonitrile (J.T. Baker) in gradient (A: 99.5–80.0%), and a flow rate of 0.2 mL/min. The retention time was approximately 5.9 min and the total run time was equal to 13 min. The mass spectrometer was operated in positive electrospray ionization (ESI). The capillary and cone energies were set at 1.2 kV and 18 V, respectively. The ESI source temperature was maintained at 110 °C and the desolvation temperature was kept at 350 °C. The collision energy was equal to 12 eV for both 8-hydroxy-2’-deoxyguanosine and [15N5]-8-hydroxy-2’-deoxyguanosine. The limit of detection (LOD) and the limit of quantification (LOQ) were used to estimate the sensitivity of the method, and equaled 0.4 and 1.2 nmol/L in the urine, respectively. The LOD was determined as the lowest concentration of a detected analyte, with at least a 3:1 signal-to-noise ratio, and the LOQ was defined as the concentration of an analyte that has at least a 10:1 signal-to-noise ratio. The average recovery was 100.6% and the average precision of the method was 5.31%.

The serum concentrations of IL-6 (pg/mL) were measured by an enzyme-linked immunosorbent assay (ELISA) according to the manufacturer’s recommendations (DRG Diagnostic, Marburg, Germany). The absorbance was measured using a plate reader (Anthos Zenyth 200 rt, Biochrom, UK). Precision: Intra assay coefficient of variation 4.2%, inter assay coefficient of variation 4.4%.

Serum lactate dehydrogenase (LDH) and creatine kinase (CK) activities were measured by kinetic methods using commercial kits (Bio-mar cat. number 41220 and 1001055, respectively) and an Evolution 300 UV–Vis spectrophotometer (Thermo Fisher Scientific, Waltham, MA, Madison, WI, USA) with a Peltier water-cooled cell-changer.

### 2.6. Statistical Analysis

All data were analyzed using the STATISTICA version 13.1 software for Windows (StatSoft, Tulsa, OK, USA). The quantitative variables are presented using the arithmetic mean and standard deviation for data with normal distribution (verification by the Shapiro–Wilk test), and in the absence of normal distribution, using the median and the minimum and maximum values. To compare the obtained results between subsequent stages, an ANOVA test for repeated measurements (for data with normal distribution) or a Wilcoxon test (for data without normal distribution) was used. Pearson correlation coefficients were performed to determine the relationship between the analyzed parameters (and data without normal distribution were log transformed). The change (Δ) represents stage ID minus stage B measurements or stage ID&BEET minus stage ID measurements. Statistical significance was accepted when *p* < 0.05. Statistical power of the obtained results (exogenous and endogenous antioxidants, biomarkers of inflammation, oxidative, and skeletal muscle damage) is in Supplementary Materials (Table S2).

## 3. Results

### 3.1. Anthropometric Parameters, Physical Activity Level, and Maximum Rate of Oxygen Uptake

In both groups of fencers, there were no significant changes between the consecutive stages of the study in such parameters as mean body weight, FM%, FFM%, or physical activity level. Significant changes were only observed in the mean values of the maximum rate of oxygen uptake, which was higher after ID&BEET in comparison to ID (Table 1). Fencers before this test at stage B and after stage ID consumed a meal with maltodextrin, but after stage ID&BEET, the meal served before the test consisted of beetroot juice. A comparison of the overall characteristics of the group of women to the group of men shows that in all stages, the body weight and FFM% of the women were significantly lower than that of the men, and, additionally, at stage B, such parameters as PA and VO_2max_ were lower, but after stages ID and ID&BEET, such differences were not observed. Despite the significant differences in FM% between men and women, the body fat mass of both the women and the men was similar (Table 1).

### 3.2. Energy and Nutrient Content in the Diet

In the studied groups of women and men, there were no differences in energy intake (per kilogram of body weight) relative to the macronutrient intake, or in the selected nutrient intake in relation to the reference range. Therefore, these data are presented without division between women and men (Table 2). The analysis of differences in the selected nutrient intake during consecutive stages shows that significant changes were only observed in the intake of protein, expressed as energy percent and relative to the intake (percentage of the reference range) of vitamin B_12_ during stages ID and ID&BEET. The mean or median values of the vitamin and mineral intake in the case of most nutrients were higher than the recommended dietary allowance or adequate intake. Only in the cases of folate (in all stages) and calcium (at stage B and during stage ID&BEET) was the intake lower than the reference range. For the intake of β-carotene, the reference range was been determined. The daily intake (as a median value and range in µg/day) of this nutrient in the consecutive stages of the study was as follows: 4399.3 (1991.2–23,568.6), 4584.5 (2678.4–12,379.3), and 4672.1 (1315.3–12,404.7), respectively. There were no significant changes between the dietary intake of β-carotene in the subsequent stages.

### 3.3. Biomarkers of Oxidative Stress, Inflammation, and Skeletal Muscle Damage

The profiles of serum antioxidant levels as well as the biomarkers of oxidative, inflammation, and skeletal muscle damage in the studied group of fencers are presented in Table 3. There were no significant differences between females versus males with regard to any characteristic, except for serum CK activity (women: Stages B, ID, and ID&BEET activity was as follows: 61.90 ± 28.65, 70.20 ± 27.30, and 83.70 ± 42.11 U/L, respectively; men: 149.10 ± 115.50, 120.90 ± 58.82, and 175.80 ± 92.64 U/L, respectively; and the *p*-values between these groups of fencers were 0.032, 0.024, 0.010, respectively) and serum beta-carotene concentration after stages ID and ID&BEET (women: The serum concentration after stages ID and ID&BEET was as follows: 0.385 ± 0.113 and 0.329 ± 0.098 µmol/L, respectively; men: 0.229 ± 0.088 and 0.218 ± 0.107 µmol/L, respectively; and the *p*-values between these groups were 0.003 and 0.026, respectively). The analysis of variance with repeated measures showed significant differences in CK serum activity only in men between stages ID and ID&BEET (*p* = 0.038). Since only these two parameters were significantly different between women and men, the rest of the variables were analyzed together in all fencers (n = 20) (Table 3.) Detailed results from the group of women and men are in the Supplementary Materials (Table S3). In the analyzed exogenous antioxidants, significant changes were observed only in the serum concentration of selenium— a significant decrease after stage ID versus stage B, but a significant increase after stage ID&BEET versus stage ID. Among the endogenous antioxidants, a significant elevation at stage B as compared to stage ID for the activity of GPx1 and GPx3 was observed. Additionally, GPx1 activity was significantly higher after stage ID&BEET versus stage ID. In the group of parameters describing oxidative damage, only the serum concentration of MDA after stage ID&BEET was significantly higher than after stage ID. Of the parameters describing skeletal muscle damage, a significant elevation in the serum activity of LDH after stage ID in comparison to stage B was observed; however, after stage ID&BEET, the mean value of this parameter did not change significantly in comparison to the value after stage ID. There were no significant differences between the consecutive stages with regard to any characteristic, except for those mentioned above. 

### 3.4. Relationship Between the Analyzed Parameters

Apart from the changes in the absolute values of the parameters between the consecutive stages of this study, a number of relationships between the anthropometric parameters, physical activity levels, and values of the maximum rate of oxygen uptake, and the serum concentration/activity of antioxidants, the biomarkers of inflammation, and oxidative and skeletal muscle damage were also observed (Table 4).

After both stages ID and ID&BEET, significant positive linear correlations were noted between the following parameters: VO_2max_ versus PA, FFM% versus VO_2max_, ∆LDH versus ∆MDA, vitamin A versus vitamin E, and ∆vitamin A versus ∆vitamin E; meanwhile, negative correlations were observed between FFM% versus β-carotene and LDH versus β-carotene. The same level of relationships as those for FFM% was also observed for FM%, only with this kind of difference, the direction of change was the opposite.

**Figure 3:**
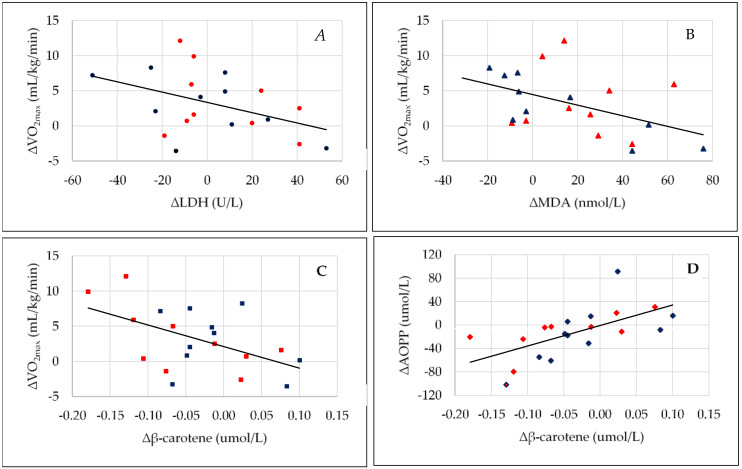
(**A**–**D**). The relationship between changes of VO2max and changes of: LDH (**A**), MDA (**B**), and β-carotene (**C**), (r = −0.518, *p* = 0.028; r = −0.472, *p* = 0.036; r = −0.500, *p* = 0.029—respectively) as well as changes of β-carotene with changes of AOPP (**D**) (r = –0.500, *p* = 0.029). Red markers—women, blue markers—men. Changes were calculated as the difference between the absolute values after ID&BEET (implementation of dietary recommendations and beetroot juice supplementation) minus the values after ID (implementation of dietary recommendations). VO2max: Maximum rate of oxygen uptake; MDA: Malondialdehyde; LDH: Lactate dehydrogenase; AOPP: Advanced oxidation protein products.

Additionally, after stage ID, positive correlations between the following parameters were observed: GPx3 activity versus selenium concentration, GPx3 activity versus AOPP level, CP activity versus cooper level, and negative CP activity versus AOPP concentration. Furthermore, after stage ID&BEET, negative exploratory correlations were noted between ∆VO_2max_ versus ∆LDH activity (Figure 3A), ∆VO_2max_ versus ∆MDA concentration (Figure 3B), ∆VO_2max_ versus ∆β-carotene level (Figure 3C), and ∆CP activity versus ∆IL-6 concentration; meanwhile, positive correlations were observed between: ∆β-carotene level versus ∆AOPP level (Figure 3D), FFM% versus LDH activity, ∆PA versus ∆GPx3 activity, LDH activity versus CK activity, GPx3 activity versus MDA level, ∆LDH activity versus ∆GPx3 activity, and ∆LDH activity versus ∆GPx1 activity (Table 4).

## 4. Discussion

This is the first well-controlled intervention study presenting differences between the changes of the biomarkers of oxidative stress, inflammation, and muscle damage associated with the use of diet and long-term beetroot juice supplementation in a group of elite fencers. In two consecutive stages of the study, based on the implementation of dietary recommendations with and without beetroot juice supplementation, there were no significant changes in the mean values of body weight, body composition, physical activity level measured by MET, or nutrient intake in relation to the reference range, except for small differences in vitamin B_12_ and the percentage of energy from protein. The vitamin and mineral intake was at the recommended level or, in the some cases, exceeded said level. Despite this, after four weeks of beetroot juice supplementation, the mean value of the maximum rate of oxygen uptake performed after consumption of a meal with this juice was significantly higher in comparison to the value at stage B and after stage ID (at these times, for the meal before the maximum rate of oxygen uptake was measured, maltodextrin was added). In regard to the parameters measured in the serum, erythrocytes, and urine, significant changes in the serum concentration of selenium and GPx1 activity (after stages ID and ID&BEET ), GPx3 and LDH activities (after stage ID), and MDA level (after stage ID&BEET ) were observed. A deeper analysis of the relationship between the absolute values of the measured parameters and their changes revealed many interesting results, which illustrated the complexity of the changes that occurred in the body during the applied interventions.

After both stages ID and ID&BEET, we observed a statistically significant negative correlation between FM% and the absolute values of VO_2max_, and an opposite relationship with FFM%. These correlations have also been confirmed by other studies, which have found that, on the one hand, a high FFM% connected to a high muscle mass (the main component of FFM) resulted in increased VO_2max_ [60,61], and, on the other hand, persons with a high FM% displayed significantly lower values of VO_2max_ [60,61,62].

Moreover, after both stages, ∆LDH correlated positively with ∆MDA, which indicates that in fencers, the serum concentration of MDA increased in parallel with the increase in training intensity. Confirmation of this assumption can be found in the results of other studies, in which MDA levels during exercise were positively correlated with muscle damage [63] and were dependent on exercise intensity (64,65). In our study, we observed that the mean serum concentration of MDA increased significantly after stage ID&BEET, but this effect was not observed after stage ID. Bearing in mind the results of the studies conducted by Lovlin [64] and Moflehi [65], it can be assumed that the net intensity training studied by us in the fencers during stage ID was lower than that during stage ID&BEET, which was difficult to capture using MET in order to assess the level of physical activity. In regard to the serum concentration of MDA observed in our study, the opposite effect of beetroot juice supplementation was observed by Hasibun [66]. The authors measured the serum concentration of MDA in young sports science students after maximal physical activity by performing a bleep test before and after one month of the training program with moderate intensity. The athletes were divided into two groups: A treatment group with beetroot juice supplementation (300 ml/day) and control group without juice. After one month, the serum concentration of MDA in the training group given beetroot juice compared to the control group was significantly lower. These discrepancies between our results and those noted by Hasibun [66] may be connected not only to differences in exercise intensity, but also to other factors such as training level and physical activity level within a few days before blood sampling, as evidenced by the results of other studies [67,68]. Similar changes over time after exercise have also been observed in the serum activity of LDH and CK. Moreover, the level of increase was also dependent on exercise intensity and training level. The increased serum concentrations of LDH, as well as of CK, are used as an indicator of the damage of muscle membrane and other tissue structures [69]. Both resistance [70] and aerobic exercise [71] promotes CK and LDH changes that increase muscle damage after an exercise session. In a study with recreationally trained men, the responses of CK and LDH after performing different resistance and aerobic exercise protocols were investigated—aerobic training at 60% and 80% VO_2max_, a resistance exercise session with a bi-set protocol, and a resistance session with multiple sets of protocols. Blood samples were collected before, immediately after, and 24 h following the experimental protocols. After 24 h, there was a significant increase in CK for the 80% and 60% VO_2max_ protocols vs. baseline, but in the case of LDH, a significant increase after 24 h was observed only after aerobic training at 80% VO_2max_ [72]. Moreover, exercise modality can affect the serum concentration of CK, which is related to the magnitude of eccentric contractions during activity and the following range of muscle disruption [73]. After an eccentric resistance training, the peak of the serum levels of CK was observed between 72 [74] and 120 h [75]. Furthermore, a higher level of muscle cell disturbance delayed the appearance of a CK serum peak versus a lower level of disruption [73]. The abovementioned results suggest that in our study, the fencers during stage ID&BEET, especially in the days just before blood sampling, engaged in a relatively high-intensity training, which could have been the result of improved physical performance associated with the consumption of beetroot juice. The group of highly trained fencers in the present study could not refrain from exercising for a few days before each stage of the study, as this would have conflicted with their training schedules.

In our study, only after stage ID&BEET were changes in VO_2max_ correlated negatively with changes in MDA and LDH. This suggests that an increase in VO_2max_ after beetroot juice supplementation is dependent on the level of these parameters before the physical activity test. This is very well visible in Figure 3A,B. Despite the significant increase in VO_2max_ after stage ID&BEET versus stage ID, this increase was not observed in all fencers. The highest positive changes of this parameter occurred in the fencers who had a decrease in LDH and MDA. These relationships indicate that the important determinants of the effectiveness of beetroot juice, in the aspect of improving physical performance, are the serum concentrations of LDH and MDA. As mentioned above, higher levels of LDH and MDA are connected to high-intensity training, and in the fencers in this study, the net effect of beetroot juice was smaller—and maybe even abolished—by the fatigue associated with intense training. Exercise and skeletal muscle fatigue are complex processes with numerous potential causes, ranging from the central nervous system function and decreased pH to the activity of myosin at the individual cross-bridge level [76]. Intense muscle contractions or exercise contribute significantly to a decrease in pH within the active muscles and in the whole body, and likely have some negative effect on exercise performance [77]. Acidic conditions are also connected to oxygen separation from hemoglobin. Many years ago, it was shown that adding hydrogen ions to blood reduces the oxygen-binding affinity of hemoglobin [78]. This means that pH can modulate the oxyhemoglobin dissociation profile, and, for a given oxygen pressure, acidosis results in a reduction in oxygen saturation [79]. These factors may explain the reason why the increase in VO_2max_ after the consumption of beetroot juice was not of a similar level in all of the studied fencers.

Additionally, very interesting relationships were observed between the serum concentration of β-carotene versus FFM% and LDH after both stages (ID and ID&BEET), and, moreover after stage ID&BEET between ∆β-carotene and both ∆VO_2max_ and ∆AOPP. In the general population, it has been shown that the serum concentration of carotenoids is negatively related, among others to FM% and positively to FFM% [80,81] as well as the serum concentration of triglycerides [82]. In the elite fencers in this study, FM% was much lower than in the general population, and this may be one factor related to the opposite relationship between the serum concentrations of β-carotene and FFM% obtained by us. The second factor may be high-intensity training, which is connected to the redistribution of some antioxidant vitamins between the tissue and plasma. β-carotene in the body is located in the blood (i.e., the membranes of circulating cells and lipoproteins) and in tissue (i.e., the membranes of cells and nucleus) [83]. For example, in an animal study, after endurance training, a decrease in the concentration of vitamin E in skeletal muscle, liver, and heart has been shown. The authors suggest that the reduction of tissue vitamin E after training probably reflects the increased free radical production at the mitochondrial inner membrane during exercise, which can enhance the chance of free radical leakage, causing detrimental chain reactions and leakage of vitamins from cells into the extracellular space and then into the blood [84]. It seems that the mechanism of blood carotene increase due to intense exercise may be similar to that connected to the higher serum activity of LDH and CK. This may be confirmed by the positive relationship between ∆β-carotene and ∆AOPP after stage ID&BEET observed in our study. This can also explain the negative relationship between ∆VO_2max_ and such parameters as ∆ β-carotene, ∆LDH, and ∆MDA observed after stage ID&BEET. Despite the abovementioned observed changes, no significant changes in the concentration of urine 8-oxodG were observed, which means that in both stages, there was no significant DNA damage, thus considered a very beneficial effect.

After both stages ID and ID&BEET, a significant increase in GPx1 activity was observed, as well as fluctuations of the serum concentration of Se and, additionally, after stage ID, a higher concentration of GPx3 activity. However, only after stage ID&BEET was the serum concentration of MDA positively correlated with GPx3 activity, and also such positive interdependence as ∆PA with ∆GPx3 activity, as well as ∆LDH activity with both ∆GPx1 and ∆GPx3 activities. These interdependences indicate a specific effect observed after stage ID&BEET—an increase in GPx1 and GPx3 activities occurred only in those fencers who had an increase in serum LDH activity and PA. In fencers in which LDH activity increased, the activity of both GPx1 and GPx3 also increased. Studies both in animals and in humans have shown that GPx activity increases in blood or in tissues after aerobic exercise [85,86]. This adaptation seems to be specific to muscular fibers, which are the main source of free radical production during exercise, and to training intensity. Powers examined the influence of exercise intensity and duration (exercise for four days/week on a motor-driven treadmill for 10 weeks) on antioxidant enzyme activity in locomotor muscles differing in fiber-type composition [87]. The applied training induced significant increases in GPx activity only in red gastrocnemius muscle fibers, and the magnitude of the GPx increase was directly related to exercise duration. In another study, the effect of endurance training on GPx activity was investigated in the skeletal muscle, heart, and liver of female rats after ten weeks of treadmill training. Trained rats showed a 62% higher GPx activity only in deep vastus lateralis muscle compared to control rats, but in the soleus, GPx activity remained unchanged [88]. Bearing in mind training intensity, Criswell et al. showed that high-intensity interval exercise is better than moderate-intensity continuous exercise in the promotion of GPx activity in the soleus in animal study [89]. The above-described results indicate that the positive relationship observed after stage ID&BEET between both ∆LDH activity and ∆MDA level with GPx1 and GPx3 activities can be connected both with the ergogenic effect of beetroot juice supplementation and with high-intensity training of specific legs muscles, which happens during fencing training.

The impact of supplementation on GPx activity was also determined by Dehghan et al. [90]. The authors determined the effect of endurance training and cinnamon bark extract supplementation on oxidative responses induced by an exhaustive exercise schedule in rats. Regular training increased GPx activity in the trained, supplemented, and exercised group, but GPx activity was not affected by the cinnamon bark extract treatment in untrained rats. These results suggest that additional use of regular training and antioxidant extract supplementation protect healthy male rats against oxidative damage induced by exhaustive exercise. Cases et al. found an elevation in GPx activity in lymphocytes after a single bicycle ride or after swimming, and the authors implied that oxidative stress and the necessity of protection against oxidative damage may be partially responsible for the elevation in the activity of these enzymes as a consequence of exercise [91]. Elosua et al. also reported that the activity of extracellular GPx rose from the baseline level after an acute bout of aerobic exercise, but 1 h later, during rest, the enzymes’ activities fell back to basal levels [92]. However, the enzymes’ activities were again increased 24 h later. In our study, the relationship between ∆LDH and ∆GPx3 activities observed after stage ID&BEET suggest that higher intensity training connected to higher LDH activity can induce an antioxidative enzyme synthesis path.

After both stages ID and ID&BEET, the median value of the serum concentration of IL-6 in the fasting stage did not change significantly compared to the value at stage B. Our results are consistent with two other studies. Clifford et al. analyzed the plasma concentration of IL-6 in recreationally active males after consumption of a high dose of beetroot juice, a lower dose of beetroot juice, or an isocaloric placebo immediately, 24 and 48 h after the completion of 100 drop jumps [93]. In all the studied groups, there were no significant changes between the pre-exercise plasma concentration of IL-6 and both after 24 and 48 h post-exercise. The main effects observed for Il-6 were connected to a significant increase immediately and 2 h post-exercise, but returned to baseline values by 24 h post-exercise, and no group interaction effects were detected. The same effect was observed after cherry juice consumption in marathon runners. Both in the placebo group and in the group with cherry juice consumption, there were no significant differences in the serum concentration of IL-6 at baseline and after the 24 h post-running test, despite that immediately after the effort, IL-6 was significantly higher in comparison to the baseline values, but immediately after the race, the serum concentration of IL-6 in the cherry juice group was significantly lower than in the placebo group [94]. However, a beneficial effect after long-term treatment with betalain-rich redbeet extract was observed by Pietrzkowski et al. in a study with participants experiencing osteoarthritis symptoms. The authors found that the serum levels of AOPP were reduced by up to 48% after 10 days of the treatment, and, in addition, this kind of treatment also resulted in the reduction of the blood level of IL-6 by 22–28% [95]. These discrepancies between the abovementioned studies and our results may be connected to the fact that a disease such as osteoarthritis is connected to a permanent increase in inflammation parameters, whereas in the case of intense physical exertion, an increase in the concentration of inflammatory markers is transient. Although, the results of these studies suggest that beetroot nutrients have an anti-inflammatory potential, and the observed effects are dependent on the type of factors causing them.

In our study, after stage ID, we observed a positive relationship for serum CP activity versus copper concentration, and this result is consistent with other studies. For example, Twomey et al. observed that the serum concentration of copper was positively related to CP, but this interdependence showed a significant dispersion around the population regression line. Differences in the total concentrations of copper of about 8.0 mol/L can occur in healthy patients at a given CP concentration [96]. Additionally, after stage ID, we observed a negative relationship between serum CP activity versus the concentration of AOPP, which indicates a protective effect of this antioxidant. CP is known to be an acute phase reactant protein that also acts as a ferroxidase, and therefore indirectly decreases the generation of the reactive oxygen species hydroxyl radical [97]. This multifunctional protein was dubbed by Bielli and Calabrese as a ‘‘moonlighting protein’’ [98]. Surina-Marysheva et al. showed the very important effect of CP on the number and resistance of erythrocytes during acute physical exercise [99]. In a study with rats, the administration of CP 24 h before acute physical exercise normalized membrane resistance in red blood cells and maintained the integrity and functional properties of erythrocytes during acute physical exercise, which is connected to severe hypoxia, hemodynamic stroke, blood concentration, and increased coagulation of the blood. The CP-induced increase in the resistance of erythrocytes to osmotic and acid treatment and stabilization of erythrocyte membranes was probably related to the pleiotropic properties of this protein. The authors concluded that CP has a direct antioxidant effect, which normalizes the oxidation potential in the liquid part of the blood and cell membranes. These properties of CP may be connected to the negative relationship observed by us between CP an AOPP. In contrast to IL-6, whose serum concentration after acute exercise drops relatively fast, a higher serum concentration of CP persists for a much longer time, which may provide a longer beneficial antioxidant effect connected to a decrease in the serum concentration of AOPP. For example, after long-distance running, the serum concentration of CP was higher after one, two, and four days of training in comparison to the resting value but after seven days was lower than at baseline [100]. In the trained runners during the six-week training period (mean weekly training distance was 62.0 km), in the blood samples collected in the morning 48 h after the last race or bout of training, the mean serum concentration of CP was higher than in the control group [101].

Despite our efforts, our study has some limitations such as: Small number of the study subjects which was also connected with lack of randomization, using indirect markers of muscle damage and lack of serum concentrations of nitrates and nitrites. With respect to the issue of the study group size, I would like to emphasize that our research included the elite of fencers—the national team and that is a very small group of people. Extending this group would make the study group heterogeneous, with a varying training level as well as training load with respect to both quantity and quality. Nevertheless, we believe that our findings add to current knowledge and aid the design of customized strategies related to oxidative stress and beetroot juice supplementation.

## 5. Conclusions

Our study involved a deep analysis of the changes in oxidative stress, inflammation, and muscle damage markers, as well as of the relationship between the analyzed parameters after four weeks of implementation of dietary recommendations, and after that, four weeks of implementation of dietary recommendations with beetroot juice supplementation. Only after the stage with beetroot juice supplementation was a significant increase in VO_2max_ observed, and changes of this parameter were negatively related to changes of ∆LDH serum activity, as well as to the concentrations of ∆β-carotene and ∆MDA. Additionally, the concentration of ∆β-carotene was positively related to the serum concentration of ∆AOPP. Moreover, ∆GPx3 activity was positively related to the serum concentrations of ∆PA and ∆LDH, and, moreover, ∆GPx1 activity to ∆LDH. After the stage with implementation of dietary recommendations without beetroot juice supplementation, these relationships did not occur. These changes might have resulted from the ergogenic properties of beetroot juice and its high potential in scavenging free radicals induced by exercise. To summarize, we showed that long-term beetroot juice supplementation increases lipid peroxidation, and improvement of VO_2max_ after beetroot juice supplementation seems to be dependent on baseline LDH activity, as well as on the serum concentrations of MDA and β-carotene.

### Practical Application

Our study results suggest that changes in the ergogenic effect of beetroot juice are negatively dependent to changes in serum concentration of MDA, β-carotene, and the activity of LDH. It means that in a situation where values of these parameters increase, the ergogenic effect of beetroot juice may decrease. This practical tip can be important for both players and coaches.

## Figures and Tables

**Table 1:** Anthropometric parameters, mean physical activity level, and values of maximum rate of oxygen uptake at the studied groups of women and men in consecutive stages of the study (women, n = 10; men n = 10) ^a^.

Variable	Stages of the Study			*p ^b^*	*p* ^c^
	B	After ID	After ID&BEET		
**Women**					
Weight (kg)	63.6 ± 8.0	63.5 ± 8.3	63.6 ± 8.6	0.743	0.801
FM%	26.4 ± 4.3	25.91 ± 5.2	24.9 ± 4.8	0.303	0.059
FFM%	73.6 ± 4.3	74.09 ± 5.2	75.1 ± 4.8	0.303	0.059
FM (kg)	16.9 ± 4.0	16.5 ± 5.1	16.1 ± 4.8	0.339	0.214
FFM (kg)	46.7 ± 5.8	47.0 ± 4.9	47.5 ± 5.3	0.562	0.146
VO_2max_(mL/kg/min)	39.3 ± 4.8	39.4 ± 4.8	42.8 ± 4.7	0.668	0.011
PA	1.7 ± 0.2	1.8 ± 0.1	1.8 ± 0.1	0.325	0.544
**Men**					
Weight (kg)	82.5 ± 11.6 *	82.4 ± 11.9 *	82.7 ± 11.4 *	0.781	0.529
FM%	17.7 ± 4.7 *	16.9 ± 4.3 *	16.6 ± 4.1 *	0.123	0.584
FFM%	82.3 ± 4.7 *	83.1 ± 4.3 *	83.4 ± 4.1 *	0.123	0.584
FM (kg)	15.0 ± 4.8	14.3 ± 4.5	14.0 ± 4.3	0.068	0.505
FFM (kg)	67.6 ± 8.2 *	68.2 ± 8.5 *	68.7 ± 8.4 *	0.120	0.210
VO_2max_(mL/kg/min)	46.0 ± 7.8 *	45.3 ± 9.3	48.5 ± 10.3	0.728	0.023
PA	1.9 ± 0.3 *	1.9 ± 0.1	1.9 ± 0.1	0.088	0.659

^a^ Values are presented as mean ± SD or as median and range; *p* ^b^: B versus ID; *p* ^c^: ID versus ID&BEET; bold values denote statistical significance at the *p* < 0.05 level: * (*p* < 0.05) unpaired-sample t-test indicated significant differences between women and men at the same time of the study stage; B: Baseline parameters; ID: The first stage of the study (implementation of dietary recommendations); ID&BEET: The second stage of the study (implementation of dietary recommendations and beetroot juice supplementation); FM%: Fat mas percent; FFM%: Fat free mass percent; VO_2max_: Maximum rate of oxygen uptake; PA: Mean physical activity level.

**Table 2:** Absolute and relative energy and macronutrient intake as well as selected nutrient intake in relation to the reference range calculated from a three-day dietary records at the studied group of fencers (n = 20) ^a^.

Variable	Stages of the Study			*p* ^b^	*p* ^c^
	B	During ID	During ID&BEET		
**Absolute and relative energy and macronutrient intake**					
Energy kcal/kg bw	30.9 ± 7.4	32.6 ± 6.1	34.5 ± 6.1	0.120	0.084
Carbohydrates (%E)	45.6 ± 8.2	45.4 ± 4.2	46.6 ± 5.3	0.880	0.395
Protein (%E)	17.2 (11.3–31.2)	18.0 (14.7–30.9)	17.1 (14.1–26.2)	0.269	0.023
Fat (%E)	32.9 (26.9–52.2)	35.5 ± 3.3	35.4 ± 4.7	0.881	0.901
SFA (%E)	12.9 ± 4.5	12.7 ± 2.7	12.3 ± 2.4	0.776	0.608
MUFA (%E)	13.7 (9.0–27.5)	14.8 ± 5.0	15.0 ± 3.0	0.794	0.841
PUFA (%E)	5.49 (2.30–11.19)	5.22 (3.21–9.50)	5.17 (3.74–9.32)	0.391	0.601
**Nutrient intake in relation to the reference range**					
Vit. E (%R)	152.4 ± 50.3	148.7 ± 37.0	155.1 ± 35.6	0.746	0.573
Vit. A (%R)	148.8 ± 49.9	166.7±40.9	157.2 ± 41.4	0.367	0.913
Vit. C (%R)	148.4 ± 58.8	166.3 ± 72.2	162.8 ± 49.3	0.352	0.905
Vit. B_1_ (%R)	124.1 ± 39.3	124.4 ± 22.2	125.6 ± 24.1	0.780	0.900
Vit. B_2_ (%R)	168.8 ± 52.3	170.9 ± 34.5	168.8 ± 31.5	0.785	0.785
Vit. B_6_ (%R)	170.2 ± 58.7	184.4 ± 39.9	181.6 ± 40.8	0.262	0.827
Vit. B_12_ (%R)	182.6 (62.0–929.3)	201.7 (111.0–662.7)	177.1 (110.4–662.7)	0.526	0.020
Niacin (%R)	137.8 ± 61.2	162.0 ± 36.5	155.5 ± 40.7	0.056	0.604
Folate (%R)	93.4 ± 30.9	91.0 ± 23.1	90.9 ± 18.4	0.700	0.986
Zinc (%R)	140.5 ± 40.0	143.3 ± 34.6	141.3 ± 31.8	0.638	0.730
Iron (%R)	112.9 ± 60.0	109.1 ± 44.3	107.3 ± 40.40	0.530	0.763
Copper (%R)	154.0 (77.7–348.2)	161.4 (103.4–286.6)	160.2 (131.9–253.7)	1.000	0.496
Calcium (%R)	92.7 ± 39.0	98.9 ± 33.3	92.0 ± 21.6	0.785	0.949
Magnesium (%R)	106.8 ± 32.5	113.4 ± 25.3	112.7 ± 22.5	0.055	0.874
Sodium (%R)	249.5 ± 87.2	248.7 ± 69.3	234.6 ± 55.2	0.945	0.236
Potassium (%R)	98.6 ± 25.6	109.7 ± 21.1	110.2 ± 19.8	0.054	0.790

^a^ Values are presented as mean ± SD or as median and range; *p* ^b^: B versus ID; *p* ^c^: ID versus ID&BEET; bold values denote statistical significance at the *p* < 0.05 level; B: Baseline parameters; ID: The first stage of the study (implementation of dietary recommendations); ID&BEET: The second stage of the study (implementation of dietary recommendations and beetroot juice supplementation); bw: Body weight; SFA: Saturated fatty acids; MUFA: Monounsaturated fatty acids; PUFA: Polyunsaturated fatty acids; %E: Percent of energy; %R: Percent of reference range; Vit.: Vitamin.

**Table 3:** Exogenous and endogenous antioxidants as well as biomarkers of inflammation, oxidative, and skeletal muscle damage in fencers (n = 20) participated in the study ^a^.

Variable	Stages of the Study			*p* ^b^	*p* ^c^
	B	After ID	After ID&BEET		
Vit. E (mg/L)	10.520 ± 2.331	11.335 ± 2.469	10.912 ± 3.069	0.067	0.334
Vit. A (mg/L)	0.625 ± 0.106	0.659 ± 0.111	0.633 ± 0.130	0.138	0.251
β-carotene (µmol/L)	0.302 ± 0.135	0.307 ± 0.127	0.273 ± 0.115	0.764	0.089
Selenium (µg/L)	63.1 ± 15.2	55.3 ± 8.5	64.2 ± 13.8	0.018	0.008
Zinc (µg/L)	790.9 ± 234.9	707.6 ± 142.5	819.1 ± 204.0	0.176	0.073
Copper (µg/L)	890.5 (626.4–1689.2)	759.2 (599.8–2215.1)	786.9 (529.2–1768.0)	0.575	0.881
CP (u/L)	0.355 ± 0.161	0.426 ± 0.161	0.403 ± 0.217	0.115	0.608
GPx-1 (u/g HGB)	20.71 ± 4.54	22.99 ± 4.45	25.01 ± 3.78	0.000	0.012
GPx-3 (u/mL)	0.161 ± 0.024	0.171 ± 0.028	0.167 ± 0.023	0.006	0.233
AOPP (µmol/L)	94.02 ± 28.91	102.15 ± 34.70	89.35 ± 27.37	0.350	0.145
MDA (nmol/L)	80.52 ± 21.75	73.36 ± 16.36	90.86 ± 31.78	0.190	0.002
8-axodG (nmol/g cr)	32.49 ± 18.47	33.27 ± 18.16	33.58 ± 19.02	0.841	0.937
IL-6 (pg/mL)	4.305 (0.100–59.960)	3.215 (0.100–54.750)	3.725 (0.100–36.510)	0.438	0.679
LDH (U/L)	116.80 ± 33.38	128.25 ± 37.83	130.15 ± 33.68	0.048	0.737
CK (U/L)	75.00 (24.00–409.00)	95.55 ± 51.66	109.00 (28.00–310.00)	0.629	0.051

^a^ Values are presented as mean ± SD or as median and range; *p* ^b^: B versus ID; *p* ^c^: ID versus ID&BEET; bold values denote statistical significance at the *p* < 0.05 level; B: Baseline parameters; ID: The first stage of the study (implementation of dietary recommendations); ID&BEET: The second stage of the study (implementation of dietary recommendations and beetroot juice supplementation); Vit.: Vitamin; GPx-1: Erythrocyte glutathione peroxidase activity; HGB: Hemoglobin; GPx-3: Serum glutathione peroxidase activity; CP: Ceruloplasmin; AOPP: Advanced oxidation protein products; MDA: Malondialdehyde; 8-oxodG: 8-oxo-7.8-dihydro-2′-deoxyguanosine; cr.: Creatinine; IL-6: Interleukin 6; LDH: Lactate dehydrogenase; CK: Creatine kinase.

**Table 4:** Correlations between anthropometric parameters, physical activity level, values of maximum rate of oxygen uptake, and serum concentrations of measured parameters after consecutive stages.

Related Variables	Stage ID	Stage ID&BEET
PA versus VO_2max_ (mL/kg/min)	r = 0.692, *p* = 0.001	r = 0.632, *p* = 0.004
FFM% versus VO_2max_ (mL/kg/min)	r = 0.496, *p* = 0.026	r = 0.471, *p* = 0.042
FFM% versus LDH (U/L)	r = 0.434, *p* = 0.056	r = 0.511, *p* = 0.021
FFM% versus β-carotene (µmol/L)	r = −0.610, *p* = 0.004	r = −0.555, *p* = 0.011
LDH (U/L) versus β-carotene (µmol/L)	r = −0.468, *p* = 0.037	r = −0.479, *p* = 0.033
∆LDH (U/L) versus ∆MDA (nmol/L)	r = 0.588, *p* = 0.006	r = 0.465, *p* = 0.045
∆β-carotene (µmol/L) versus ∆VO_2max_ (mL/kg/min)	r = 0.130, *p* = 0.584	r = −0.500, *p* = 0.029
∆β-carotene (µmol/L) versus ∆AOPP (µmol/L)	r = 0.278, *p* = 0.235	r = 0.623, *p* = 0.003
∆LDH (U/L) versus ∆VO_2max_ (mL/kg/min)	r = 0.239, *p* = 0.311	r = −0.518, *p* = 0.028
∆MDA (nmol/L) versus ∆VO_2max_ (mL/kg/min)	r = 0.108, *p* = 0.651	r = −0.472, *p* = 0.036
LDH (U/L) versus CK (U/L)	r = 0.404, *p* = 0.078	r = 0.620, *p* = 0.004
∆PA versus ∆GPx-3 (U/mL)	r = −0.171, *p* = 0.472	r = 0.471, *p* = 0.036
MDA (nmol/L) versus GPx-3 (u/mL)	r = −0.033, *p* = 0.891	r = 0.447, *p* = 0.048
∆LDH (U/L) versus ∆GPx-3 (u/mL)	r = −0.011, *p* = 0.964	r = 0.600, *p* = 0.007
∆LDH (U/L) versus ∆GPx-1 (u/g HGB)	r = 0.011, *p* = 0.963	r = 0.467, *p* = 0.044
∆CP (u/L) versus ∆IL-6 (pg/mL)	r = −0.230, *p* = 0.360	r = −0.486, *p* = 0.041
GPx-3 (u/mL) versus Selenium (µg/L)	r = 0.456, *p* = 0.043	r = 0.196, *p* = 0.408
GPx-3 (u/mL) versus AOPP (µmol/L)	r = 0.528, *p* = 0.017	r = 0.223, *p* = 0.345
CP (u/L) versus Copper (µg/L)	r = 0.518, *p* = 0.019	r = 0.229, *p* = 0.332
CP (u/L) versus AOPP (µmol/L)	r = −0.445, *p* = 0.049	r = 0.195, *p* = 0.411
Vit. E (mg/L) versus Vit. A (mg/L)	r = 0.521, *p* = 0.019	r = 0.566, *p* = 0.009
∆Vit. E (mg/L) versus ∆Vit. A (mg/L)	r = 0.553, *p* = 0.011	r = 0.520, *p* = 0.019

Δ: The change represents: ID minus B measurement after the stage ID or ID&BEET minus ID measurement after the stage ID&BEET—respectively; bold values denote statistical significance at the *p* < 0.05 level; B: Baseline parameters; ID: The first stage of the study (implementation of dietary recommendations); ID&BEET: The second stage of the study (implementation of dietary recommendations and beetroot juice supplementation); FM%: Fat mas percent; FFM%: Fat free mass percent; VO_2max_: Maximum rate of oxygen uptake; PA: Mean physical activity level; Vit.: Vitamin; GPx-1: Erythrocyte glutathione peroxidase activity; HGB: Hemoglobin; GPx-3: Serum glutathione peroxidase activity; CP: Ceruloplasmin; AOPP: Advanced oxidation protein products; MDA: Malondialdehyde; 8-oxodG: 8-oxo-7.8-dihydro-2′-deoxyguanosine; cr.: Creatinine; IL-6: Interleukin 6; LDH: Lactate dehydrogenase; CK: Creatine kinase.

## Supplementary Materials

The following are available online at https://www.mdpi.com/2076-3921/9/7/571/s1. Table S1. Nutritional profile of freeze-dried beetroot juice. Table S2. Statistical power (%) of the relationship between the effect size and sample size. Table S3. Exogenous and endogenous antioxidants as well as biomarkers of inflammation, oxidative and skeletal muscle damage in women (n = 10) and men (n = 10).
